# [^68^Ga]Ga-NODAGA-RGD post MI reflects activated fibroblasts rather than angiogenesis

**DOI:** 10.1007/s00259-025-07489-4

**Published:** 2025-07-29

**Authors:** Matti Raitza, Markus Wolfien, Heiko Lemcke, Ralf Gäbel, Praveen Vasudevan, Moritz Schweins, Tobias Lindner, Markus Joksch, Anna Schildt, Jens Kurth, Änne Glass, Alper Öner, Hüseyin Ince, Felix G. Meinel, Bernd Joachim Krause, Brigitte Vollmar, Robert David, Cajetan Immanuel Lang

**Affiliations:** 1 Department of Cardiac Surgery, Rostock University Medical Center, 18057 Munich, Germany; 2 Department of Life, Light and Matter, University of Rostock, 18059 Rostock, Germany; 3 Institute of Medical Informatics and Biometry, Faculty of Medicine Carl Gustav Carus, Technische Universität Dresden, 01307 Dresden, Germany; 4 Center for Scalable Data Analytics and Artificial Intelligence (ScaDS.AI), Technische Universität Dresden, Dresden/Leipzig, Germany; 5 Rudolf-Zenker-Institute for Experimental Surgery, Rostock University Medical Center, 18057 Rostock, Germany; 6 Core Facility Multimodal Small Animal Imaging, Rostock University Medical Center, 18057 Rostock, Germany; 7 Department of Nuclear Medicine, Rostock University Medical Center, 18057 Rostock, Germany; 8 Institute for Biostatistics and Informatics in Medicine and Ageing Research, Rostock University Medical Center, 18057 Rostock, Germany; 9 Institute of Diagnostic and Interventional Radiology, Rostock University Medical Center, 18057 Rostock, Germany; 10 Department of Cardiology, Rostock University Medical Center, 18057 Rostock, Germany; 11 Department for Preventive Sports Medicine and Sports Cardiology, Technical University of Munich, School of Medicine and Health, TUM University Hospital, Munich, Germany

**Keywords:** Mouse model, Myocardial infarction, Ga-NODAGA-RGD-PET, Fibroblasts, Angiogenesis

## Abstract

**Purpose:**

Angiogenesis is crucial in myocardial healing after myocardial infarction (MI). The α_v_β_3_-integrin, a key regulator of angiogenesis, is targeted by RGD-based PET tracers like [^68^Ga]Ga-NODAGA-RGD. Yet, angiogenesis imaging using RGD-based tracers is seriously hampered by the lack of true specificity of the α_v_β_3_-integrin for angiogenic cells. Therefore, our study aimed to identify the cell type with the highest α_v_β_3_-integrin expression in the process of myocardial healing in order to determine the actual value of the PET tracer [^68^Ga]Ga-NODAGA-RGD for imaging post-MI angiogenesis.

**Methods:**

Cardiac magnetic resonance imaging (CMR) was used to assess cardiac function and morphology after 28 days in two groups: permanent ligation (PL) of the left anterior descending coronary artery and transient occlusion for 30 min (I/R). Following these measurements, hearts were excised for histological and immunohistological examinations to evaluate scar formation, capillary density, and cellular composition. PET imaging with [^68^Ga]Ga-NODAGA-RGD was conducted on day 5 and day 7 post-MI. Single-nucleus transcriptomics were performed to identify cell clusters expressing α_v_β_3_-integrin.

**Results:**

Both infarct models induced scar formation, with the PL group developing large infarcts accompanied by massive left ventricular dilation and hypertrophy of cardiomyocytes, while the I/R group exhibited small intramural scars without significant changes in LV geometry or function. PET imaging revealed significantly higher tracer accumulation in the infarct area of the PL group compared to the I/R group. Single-nucleus transcriptomics performed 5 days post-MI revealed that angiogenesis markers were enriched in the I/R group, while the highest α_v_β_3_-integrin mRNA expression was identified in the fibroblast cluster, indicating an activated phenotype.

**Conclusion:**

Activated fibroblasts are the primary target cells of [^68^Ga]Ga-NODAGA-RGD, rather than angiogenic cells. In this regard, [^68^Ga]Ga-NODAGA-RGD is most probably not a valid tracer for imaging angiogenesis during the first days post-MI.

**Supplementary Information:**

The online version contains supplementary material available at 10.1007/s00259-025-07489-4.

## Introduction

Angiogenesis plays a central role in the healing myocardium after ischemic injury as well as in tumor growth, thus representing a key mechanism of great interest for targeted therapies. The α_v_β_3_ integrin was identified as one of the key regulators of angiogenesis [1]. This finding initiated intense research on developing both pharmacological inhibitors as well as radioactive tracers binding selectively to this integrin. The cyclic pentapeptide Cyclo(-RGDfK) was the initial lead structure for the first tracers based on the high affinity and selectivity of the α_v_β_3_ integrin to this peptide [2]. Continuously refining the radiopharmaceutical´s characteristics eventually resulted in the synthesis of [^68^Ga]Ga-NODAGA-RGD. Easy and fast production of this generator-produced radionuclide and commercial availability of its precursor in cGMP quality makes [^68^Ga]Ga-NODAGA-RGD an ideal candidate for clinical translation [2, 3].

Despite the availability of excellent tracers for mapping α_v_β_3_ expression in-vivo, their clinical application in angiogenesis monitoring remains poorly established due to the lack of specificity of the integrin for this biological process. Besides endothelial cells, other cell types involved in the process of tissue healing, such as macrophages and fibroblasts, also exhibit significant α_v_β_3_ integrin expression [4, 5]. Yet, it has been widely accepted in the community, that the bulk of α_v_β_3_ integrin is found on the tips of vessels sprouting into the provisional matrix, at least in the specific setting of healing myocardium post ischemic injury [6]. This prompted us and others to allocate the RGD tracer signal from the healing tissue specifically to angiogenesis [7–9]. The impedimental unclarity concerning the specificity of the integrin for the actual underlying biological process has seriously hampered the evolution of imaging angiogenesis. Therefore, we aimed at finally settling the controversy, which cell types exhibit the highest expression level of α_v_β_3_ in the setting of healing myocardium as well as whether expression levels of this integrin represent a valuable predictor of LV remodeling.

We hypothesized that:

1. the extent of tracer accumulation is determined by the extent of myocardial damage and.
2. α_v_β_3_ integrin expression – as measured by NODAGA-RGD PET – is a marker of angiogenesis and mostly expressed on endothelial cells in the heart.

We used the cutting-edge technologies of (1) CMR to precisely describe LV remodeling in the two used infarct models as well as (2) sn-RNA sequencing of whole murine hearts to measure cell-type specific expression of α_v_β_3_ integrin. Interestingly, while our data confirm the first hypothesis, the transcriptomic data reveal that activated fibroblasts - yet not endothelial cells - show the highest expression of α_v_β_3_ integrin.

## Results

### PET based quantification of α_v_β_3_ integrin-expression within the infarction region

**Fig. 1:**
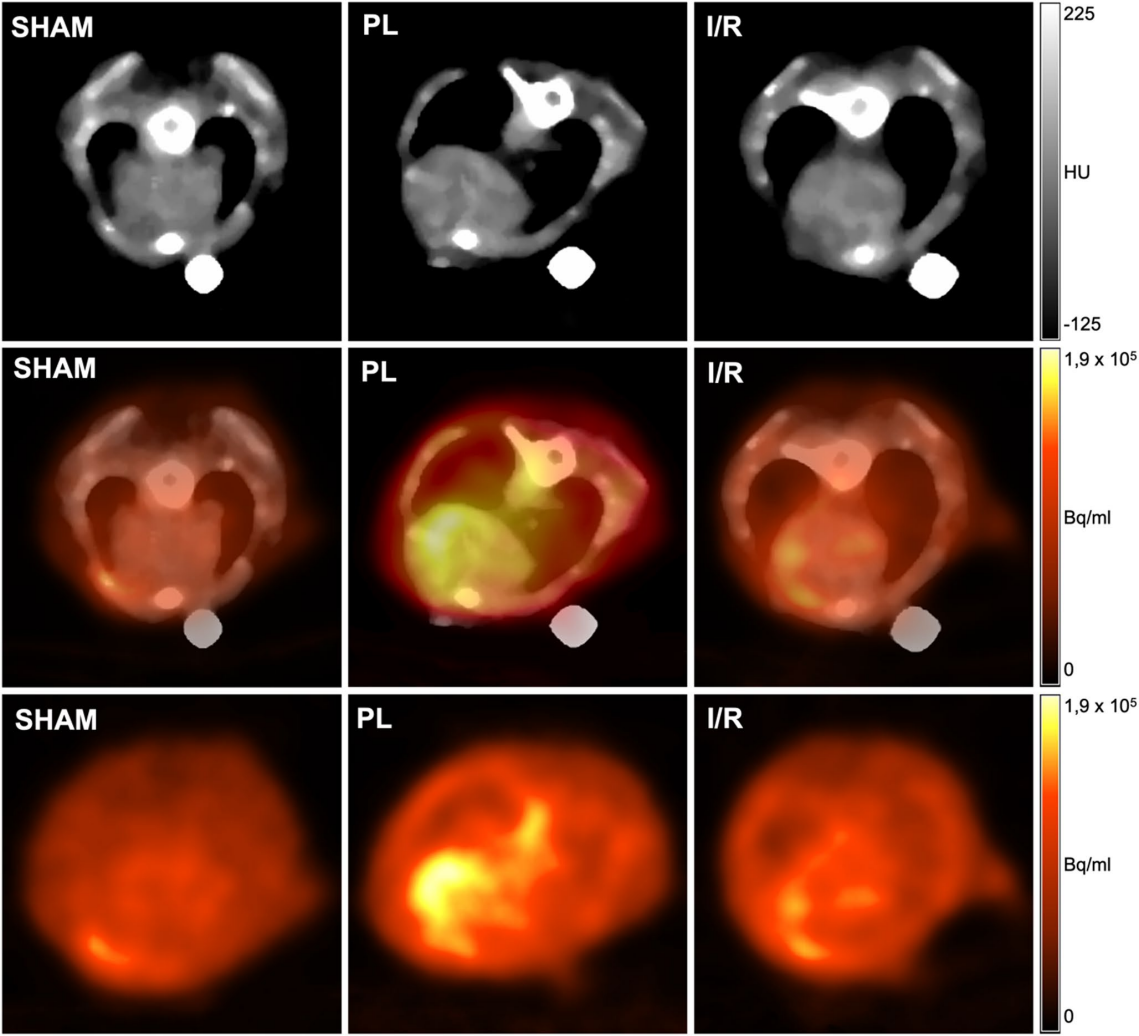
Representative axial PET/CT fusion images of the respective group on day 5 post-MI. Tracer accumulation can be clearly allocated to the infarct region in both infarct groups

**Fig. 2:**
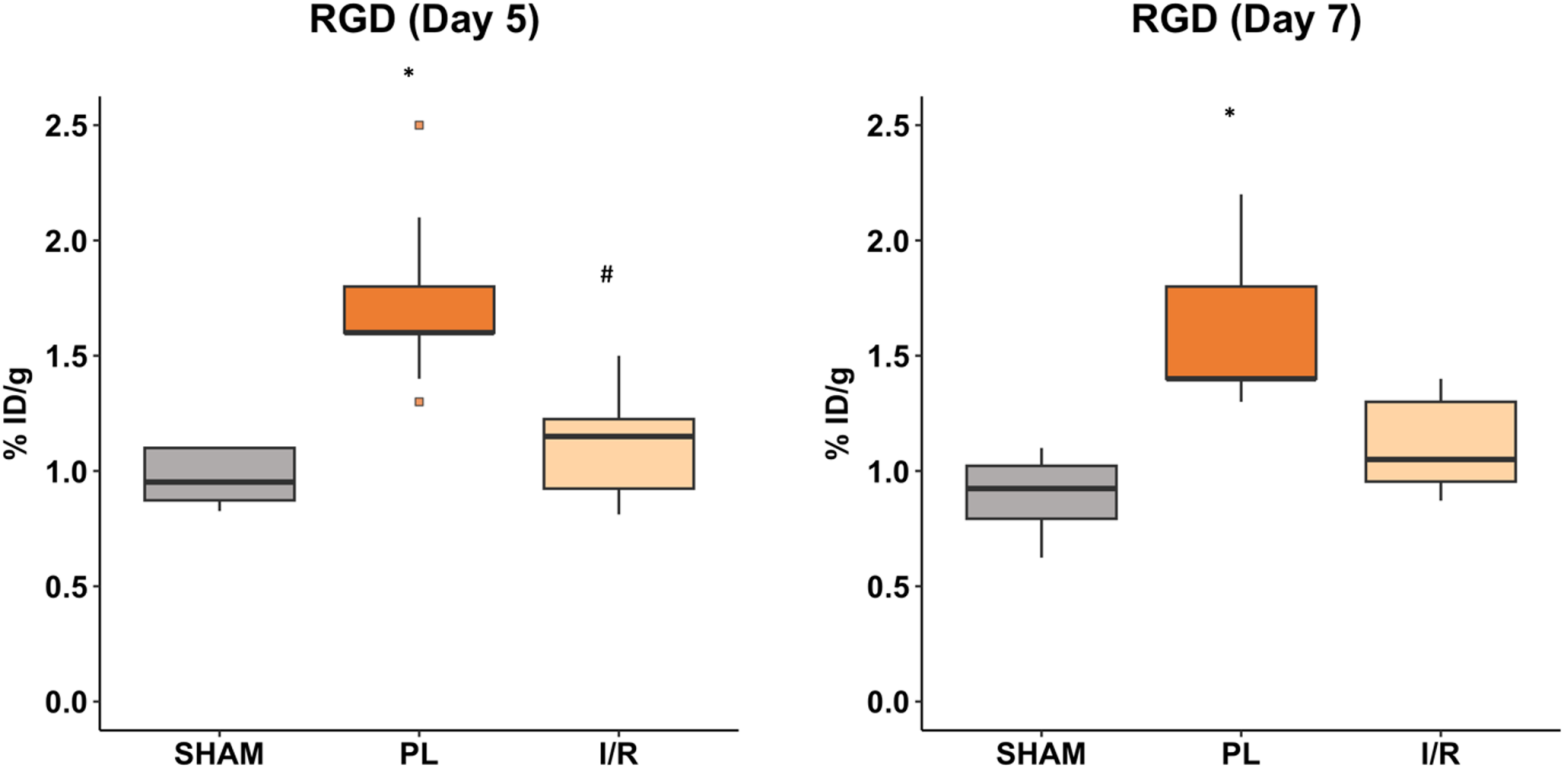
[^68^Ga]Ga-NODAGA-RGD-PET Focal tracer accumulation within the infarct area was assessed 5 and 7 days post-MI in both infarct models. %ID/g was measured by positioning a volume of interest (VOI) of 20 µL into the brightest area within the scar. Boxplots show medians (center lines), 25th and 75th percentiles (box limits), and whiskers (1.5 IQR); outliers are squares. One-way ANOVA on ranks followed by Dunn’s post hoc test was used to compare experimental groups. * *p* < 0.05 compared to SHAM group. # *p* < 0.05 compared to PL group

**Fig. 3:**
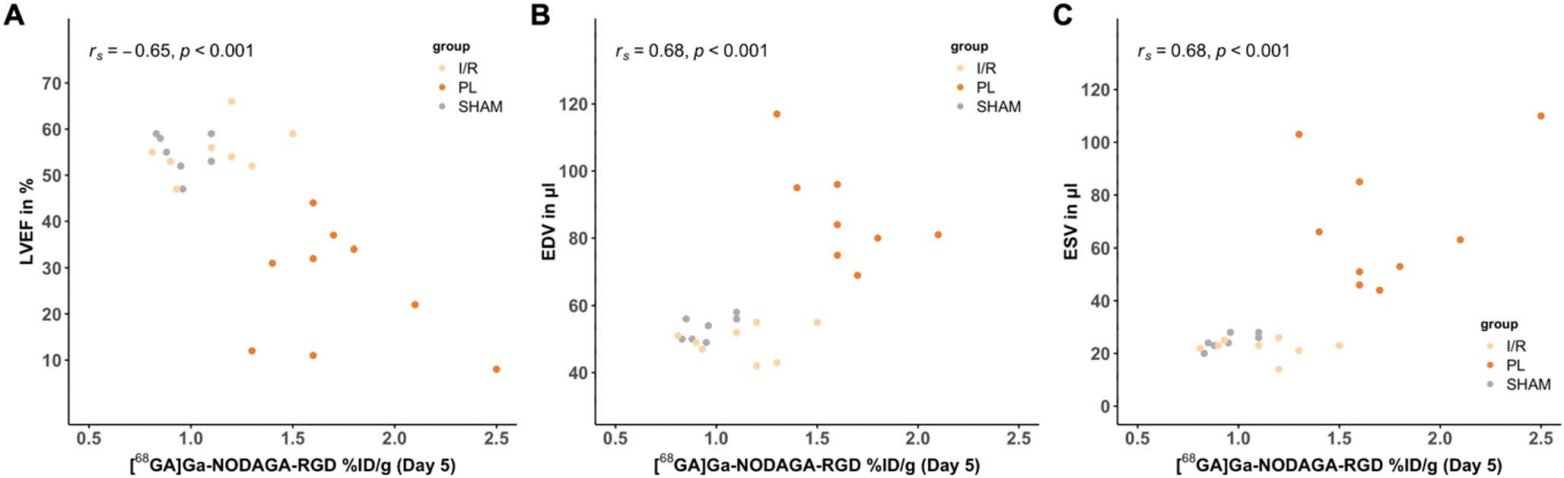
A-C Correlations between [^68^Ga]Ga-NODAGA-RGD-PET uptake at 5 days post-MI and subsequent changes at 28 days in (**A**) LVEF, (**B**) EDV, and (**C**) ESV. For correlation between tracer uptake and LVEF, EDV, and ESV EDV, Spearman´s rank coefficient was used. SHAM: *n* = 8; PL: *n* = 9; I/R: *n* = 9

[^68^Ga]Ga-NODAGA-RGD imaging was performed 5 and 7 days after MI induction. Tracer accumulation could be clearly allocated to the infarct area and was significantly higher in the PL group compared to the SHAM group at both timepoints. (Day 5: SHAM: 0.97 ± 0.12% ID/g, PL: 1.73 ± 0.37% ID/g, IR: 1.22 ± 0.36% ID/g; SHAM vs. PL: *p* < 0.001; Day 7: SHAM: 0.90 ± 0.17% ID/g, PL: 1.6 ± 0.15% ID/g, IR: 1.18 ± 0.27% ID/g; SHAM vs. PL: *p* < 0.001) (Figs. 1 and 2). Furthermore, positive correlations between EDV and ESV, and a negative correlation between LVEF, each serving as indirect metrics of myocardial damage, and tracer uptake were observed (Fig. 3A-C).

## Single-nucleus RNA-sequencing

### Fibroblasts – not endothelial cells - show highest α_v_β_3_ integrin expression

**Fig. 4:**
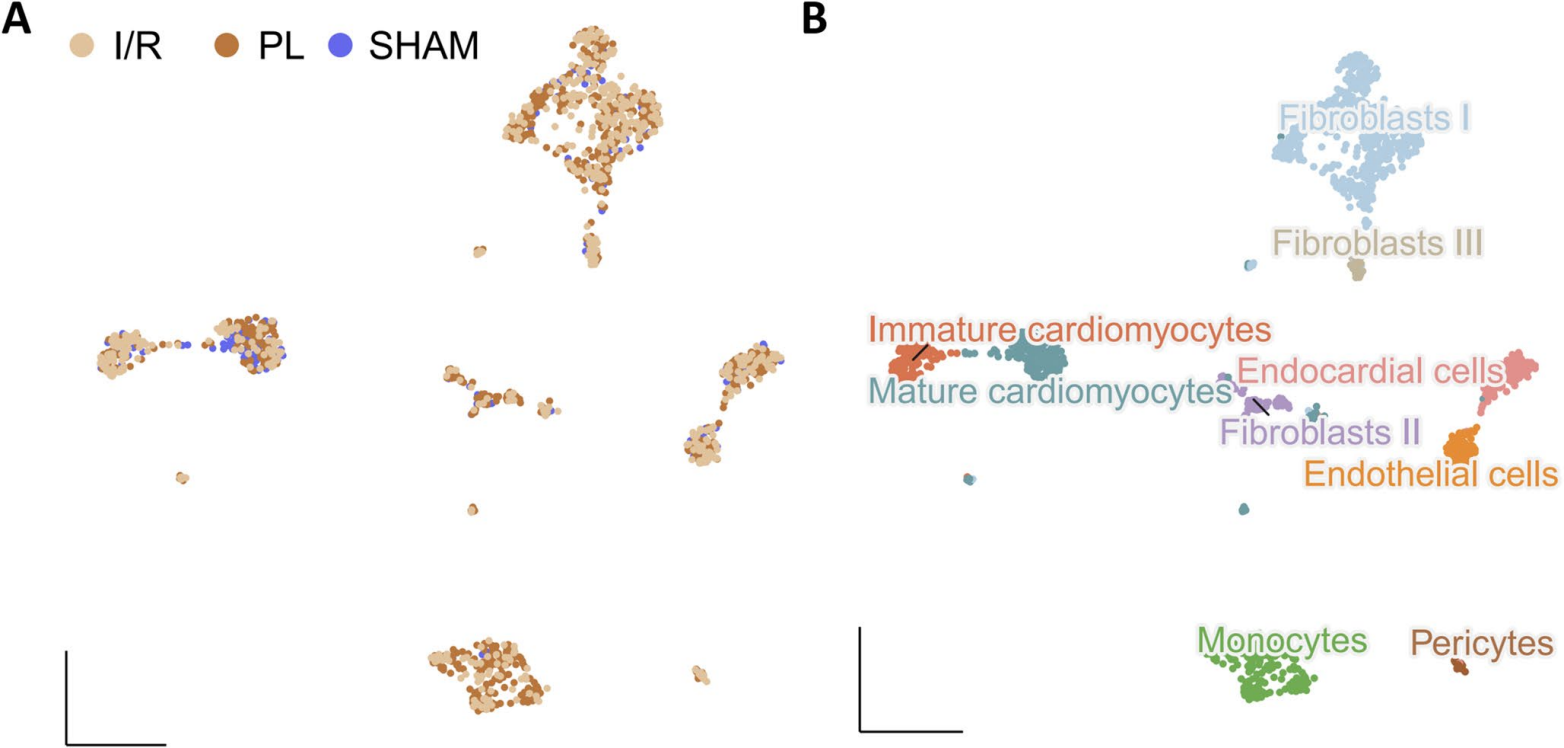
Single-nucleus transcriptome characteristics of pooled whole 129S6/SvEvTac mice hearts. **A** Integration and UMAP representation of individual PL, IR, and SHAM (*n* = 4 per group) snRNA-Seq data (5876 nuclei). **B** UMAP clustering of snRNA-seq data reveals nine distinct clusters for the indicated cell types

**Fig. 5:**
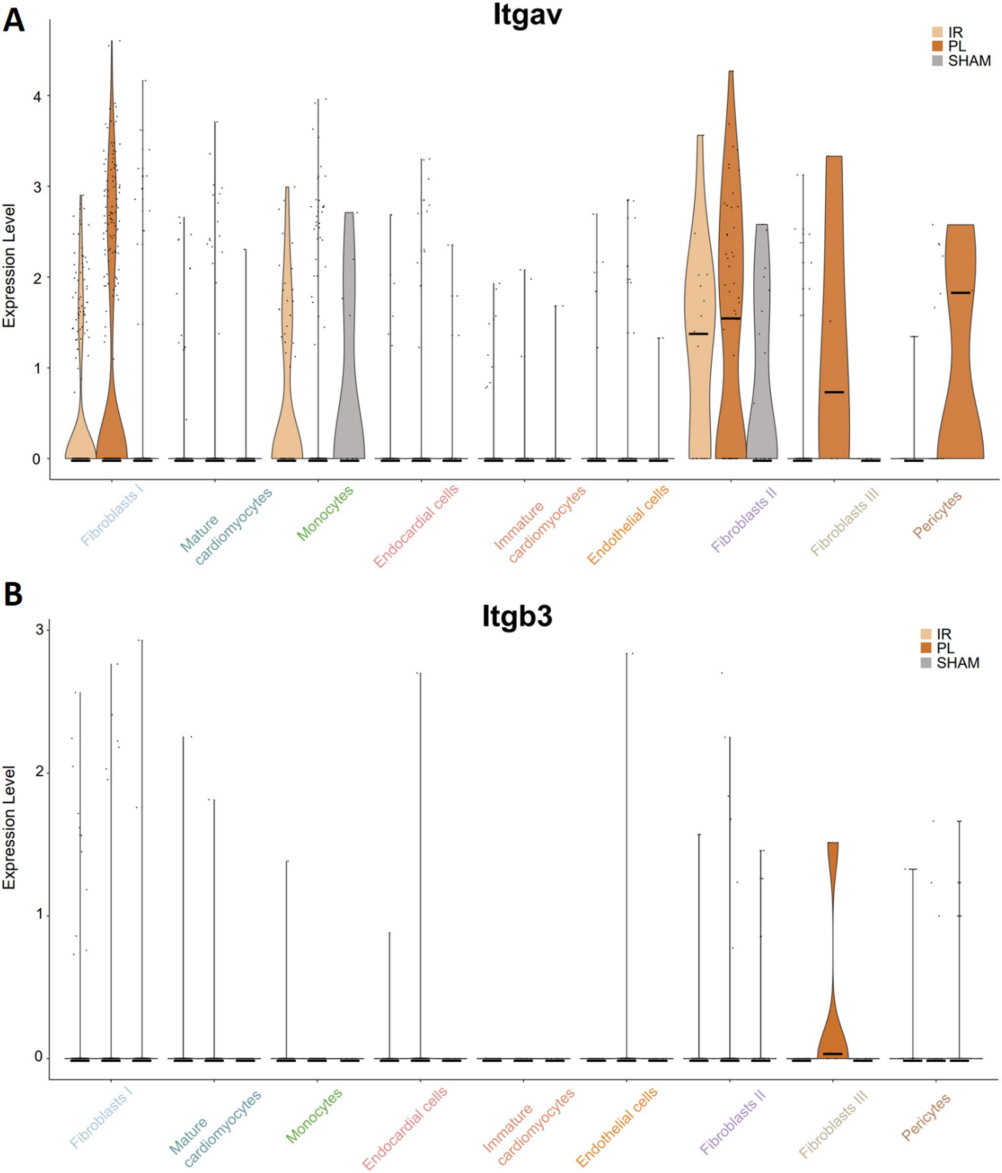
Violin plots depicting the expression the two subunits of the α_v_β_3_ Integrin: Itgav and Itgb3. Relevant co-expression of both subunits was only detected in the Fibroblast III cluster. Median values are visualized by a black horizontal bar. (*n* = 5 per group)

To unravel the controversy, if α_v_β_3_-imaging depicts proliferating endothelial cells we next performed single nucleus transcriptomics [10–13] using entire hearts of the experimental animals at day 5 post MI (Fig. 4). SnRNA-seq confirmed our finding that α_v_β_3_ expression is higher in the PL group. Yet, highest rates of α_v_β_3_ expression – the molecular target of [^68^Ga]Ga-NODAGA-RGD – are present in fibroblasts (yet not endothelial cells as postulated), hence refuting our hypothesis that α_v_β_3_ reflects particularly blood vessels sprouting into the provisional matrix of the forming scar (Fig. 5).

**Fig. 6:**
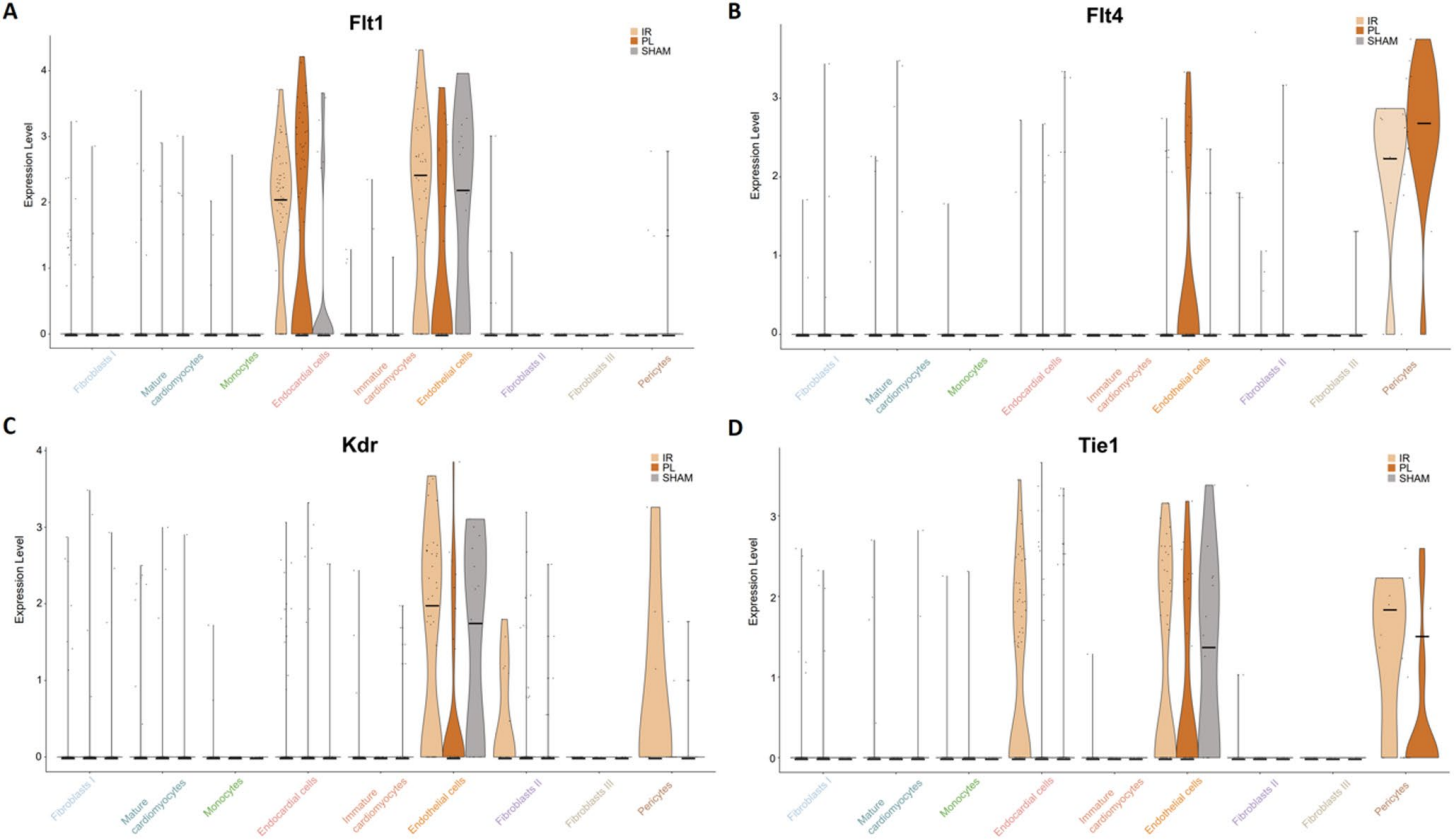
Violin plot visualization of gene markers of angiogenesis (Flt1, Flt4, Kdr, Tie1) comparing relative gene expression between the experimental groups and cell types. Median values are visualized by a black horizontal bar. (*n* = 5 per group)

Existing evidence on injury-induced angiogenesis within the infarcted myocardium is based on histological data and reports higher numbers of vessels sprouting into the injured myocardium after transient occlusion of the LAD compared to permanent occlusion within the first week post-MI [14]. In order to validate our data in the context of the existing literature, we compared the expression of well-established markers of angiogenesis (*Flt1*,* Flt4*,* Kdr*,* Tie1*) at day 5 after MI induction between I/R, PL, and SHAM [15]:

Endothelial cells expressed all these four markers at high levels. In contrast, endocardial cells showed only high expression levels of Flt1 and Tie1, whereas pericytes expressed high levels of Kdr, Flt4 and Tie1. Except for Flt4, all these angiogenesis markers were found to be higher in the I/R group compared to the PL group (Fig. 6).

### Activated fibroblasts

**Fig. 7:**
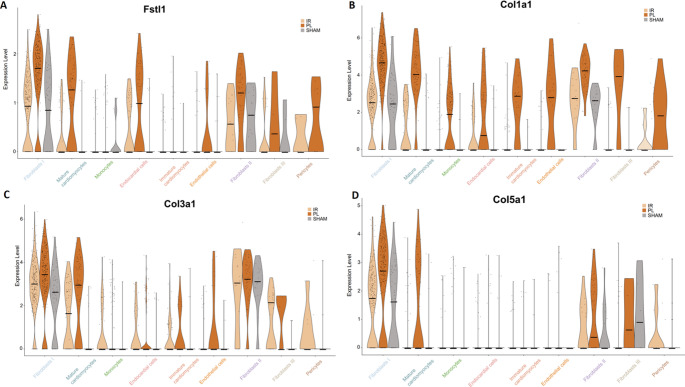
Violin-plot visualization of gene markers for injury-activated fibroblasts at day 5 post MI (Fstl1, Col1a1, Col3a1, Col5a1) comparing relative gene expression between the experimental groups and cell types. Median values are visualized by a black horizontal bar. (*n* = 5 per group)

The positive correlations of EDV and ESV, along with the negative correlation of LVEF, with [⁶⁸Ga]Ga-NODAGA-RGD uptake (Fig. 3A-C) suggest a causal relationship between the extent of myocardial damage and the level of α_v_β_3_ expression. Hence, we wondered if the bulk of α_v_β_3_ expression in the context of myocardial healing post MI may actually reflect fibroblast activation, which would be in line with the positive correlation of EDV and [^68^Ga]Ga-NODAGA-RGD uptake. As a consequence, we determined expression of markers for injury-activated fibroblasts at day 5 post MI (Fstl1, Col1a1, Col3a1, Col5a1) [16]:

In general, permanent ligation (PL) of the LAD resulted in a higher expression level of Fstl1 in all fibroblast clusters, mature cardiomyocytes, endocardial cells, endothelial cells, and pericytes compared to the I/R group (Fig. 7A). Col1a1 was expressed in higher rates in all clusters in the PL group compared to I/R. The highest expression levels have been observed in the fibroblast clusters (Fig. 7B). Fibroblasts I likewise exhibited the highest number of cells, as well as absolute levels of Col3a1 and Col5a1 expression, followed by mature cardiomyocytes and fibroblasts II (Fig. 7C-D). The PL group reveals higher expression of both collagens compared to the I/R group in each of these three cell clusters.

**Fig. 8:**
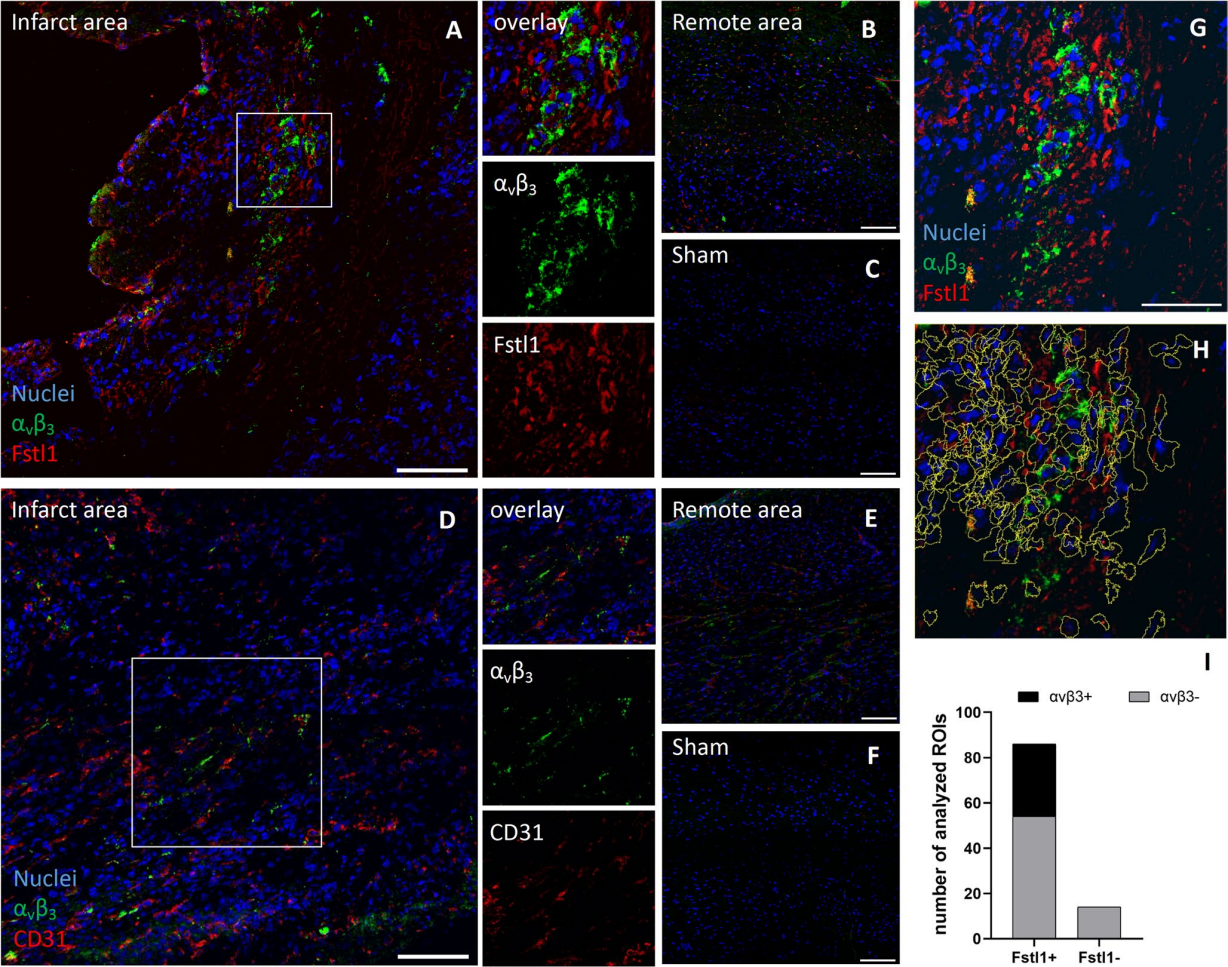
Expression pattern of α_v_β_3_-integrin, Fstl1 and CD31 in hearts at day 5 after permanent ligation of the LAD in both remote and infarcted myocardium, and the SHAM group. α_v_β_3_-integrin and Fstl1 are highly expressed within the infarct area (**A**), while profound less expression was detected in the remote area, comparable to the SHAM group (**B**, **C**). CD31 is expressed within the scar (**D**), but shows basal expression levels in the remote area (**E**) – similar to the SHAM group (**F**). Co-localization of Fstl1 and α_v_β_3_-integrin was quantified in co-labelled heart sections (**G**). Regions of Interest (ROI) were analyzed according to a positive fluorescence signal for both proteins (**H**). All α_v_β_3_-integrin positive ROIs were contained in the Fstl1-positive ROI population (**I**). Scale bar: 100 μm

Heart sections were co-labelled with antibodies targeting α_v_β_3_ integrin and Fstl1. As depicted in Fig. 8, a strong expression of α_v_β_3_ integrin was detected in the infarct area, while remote myocardium demonstrated far less fluorescence intensity for the α_v_β_3_ integrin. Almost no expression was observed in Sham operated animals.

Likewise, Fstl1 expression was found to be highly increased in the infarct zone when compared to remote myocardium and Sham control. Moreover, co-staining revealed a co-localization of α_v_β_3_ integrin and Fstl1, supporting our single-cell data.

Similarly, we performed co-labelling of CD31, a marker for endothelial cells. Compared to remote tissue, an increased fluorescence intensity of CD31 was observed in the infarct area. However, in contrast to Fstl1, co-localization between α_v_β_3_ integrin and CD31 was far less prominent.

### Effects on LV remodeling after 4 weeks

#### Global morphological and functional parameters of the left ventricle

**Fig. 9:**
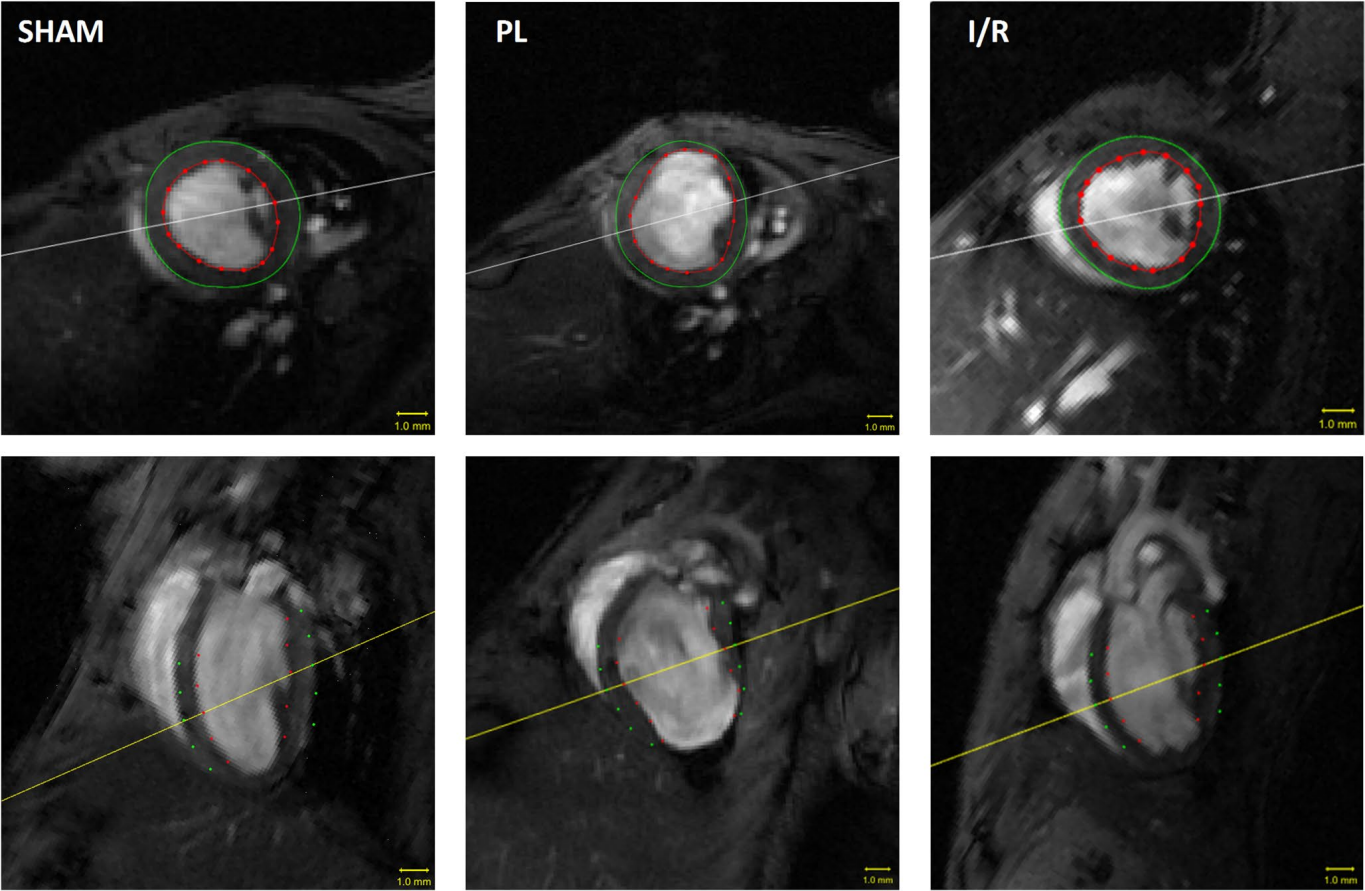
Representative end-diastolic (ED) MRI images of the left ventricle 4 weeks after SHAM, PL or I/R surgery. Top row: short axis view. Bottom row: long axis view. The epicardium is marked with green dots, the endocardium with red dots. Scale bar: 1 mm

**Fig. 10:**
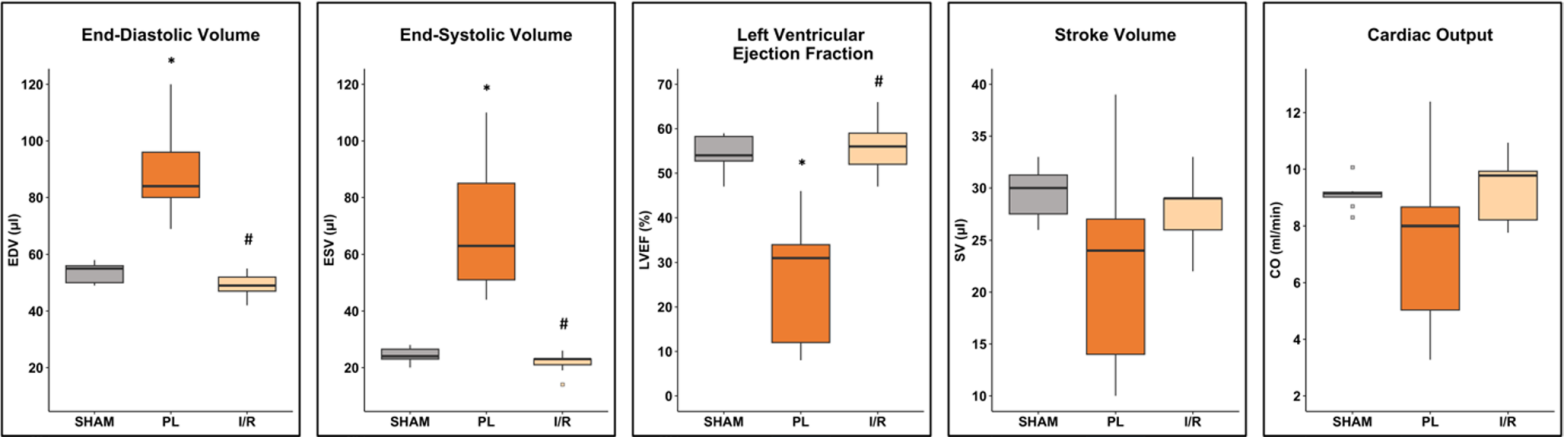
Volumetric and functional changes 4 weeks after MI induction. PL: permanent ligation of the LAD. I/R: transient occlusion of the LAD for 30 min. Boxplots show medians (center lines), 25th and 75th percentiles (box limits), and whiskers (1.5 IQR); outliers are squares. One-way ANOVA followed by Tukey’s post hoc test or one-way ANOVA on ranks followed by Dunn’s post hoc test were used to compare experimental groups. * *p* < 0.05 compared to SHAM group. # *p* < 0.05 compared to PL group. SHAM: *n* = 8; PL: *n* = 9; I/R: *n* = 9

Permanent ligation (PL) of the LAD is well known to induce a large transmural infarct, whereas transient occlusion of the LAD (I/R) results in a relatively small infarct [17]. As expected, permanent occlusion of the LAD led to massive dilation of the left ventricle (LV) with a change in chamber geometry from an elliptical to a more spherical shape (Fig. 9). In contrast, transient occlusion for 30 min had no statistically significant effect on both LV volumes and global geometry. In addition, left ventricular ejection fraction (LVEF) was only reduced in the PL group. Stroke volume (SV) and cardiac output (CO) were depressed, not reaching a level of statistical significance (Fig. 10).

#### Histological implications of LV remodeling

**Fig. 11:**
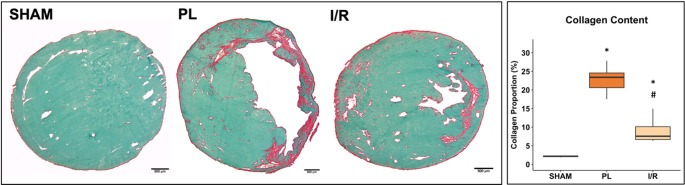
Left ventricular collagen content 4 weeks post-MI. Sirius red (collagen) and Fast Green (non-infarcted myocardium) staining of transversal shot-axis sections shows a transmural scar in the PL group and a smaller intramural scar layer in the IR group. Boxplots show medians (center lines), 25th and 75th percentiles (box limits), and whiskers (1.5 IQR). One-way ANOVA followed by Tukey’s post hoc was used to compare experimental groups. * *p* < 0.05 compared to control group. # *p* < 0.05 compared to PL group. SHAM: *n* = 4; PL: *n* = 7; I/R: *n* = 7. Scale bar: 500 μm

After the final CMR scan, hearts were excised for histological analyses. The size of the infarct scar was assessed by quantification of the cross-sectional collagen content of the left ventricle. Both infarct models induced relevant scars, with PL resulting in a significantly larger transmural scar compared to a smaller intramural scar layer on the I/R group (Fig. 11).

## Discussion

We had recently established [^68^Ga]Ga-NODAGA-RGD PET imaging for evaluation of α_v_β_3_-integrin expression within the healing myocardium in mice after experimental MI induction [7], believing in the concept that the bulk of α_v_β_3_ integrin is expressed on the tips of vessels sprouting into the provisional matrix of the healing scar [6] hence allocating the tracer signal to the biological process of angiogenesis [7–9]. Yet, evidence proving this concept had been limited and was mainly based on histological analyses.

Whereas highly specific binding of [^68^Ga]Ga-NODAGA-RGD to the α_v_β_3_-integrin could be shown in several studies [2, 18], evidence concerning the specificity of the integrin as a marker of post-MI angiogenesis remained rather scarce. When testing novel α_v_β_3_-integrin tracers, most studies typically provided solid data on specificity of the tracer and its favorable binding characteristic for in-vivo imaging [19]. However, with respect to specificity of the integrin for the biological process of post-MI angiogenesis, evidence is mostly restricted to immunohistochemical analyses, data, which have been interpreted as a close correlation of the tracer signal with staining of newly formed vessels [20].

On the other hand, expression of the integrin by other cell types, such as fibroblasts or macrophages, which play important roles in myocardial healing, have been proposed as alternative sources of α_v_β_3_-integrin expression [5, 21, 22]. This unsolved issue of specificity of the α_v_β_3_-integrin for the biological process of angiogenesis has seriously hampered clinical translation of RGD-based radiotracers in the field of myocardial healing. Hence, the ultimate goal of this study was to decipher these fundamental specificity issues in order evaluate the specific applicability of [^68^Ga]Ga-NODAGA-RGD in angiogenesis monitoring.

Here, we used the comparison of two well characterized models of experimental MI in the mouse model, namely permanent ligation of the LAD (PL) and transient occlusion of the LAD (I/R). PL is known to induce large transmural infarcts, whereas I/R induces relatively small infarct layers in the middle of the myocardium [17, 23, 24]. As expected, the PL group exhibited large infarcts with consecutive massive dilation of the LV. In contrast, occlusion of the LAD for 30 min induced a small intramural scar, without effecting global of regional parameters of both LV geometry and function.

The ischemia-reperfusion time of 30 min represents a standard in the mouse model of myocardial infarction [25]. While cardiac function assessment in wild-type animals consistently shows a decrease in LVEF to around 40% or lower after reperfusion times of 45 min or longer [17], LVEF values after 30 min range from normal [26] to approximately 40% [27]. Functional differences after 30 min of reperfusion may be attributed to strain-specific healing characteristics [28], and some extent, by different modalities used for the assessment of cardiac function.

Histology of excised hearts after the MRI scan on day 28 post-MI showed significant cardiomyocyte hypertrophy in the PL group, likewise wall thickness in the remote area was significantly higher. These findings are in line with previous studies and reflect the typical process of post-infarct LV remodeling. The positive correlation of infarct size and the extent of LV remodeling has already been described decades ago [29, 30].

Vessels sprouting into the granulation tissue within the first weeks after MI represent mainly angiogenesis. This process is defined as the sprouting of pre-existing post-capillary venules into the provisional extracellular matrix and supplies the metabolically active immature scar with energy [31]. The majority of these micro-vessels regress once the scar matures [31]. Our histological analysis of the infarct area reveals a collagen-rich scar with dramatic reduction of vessel density in the PL group, which aligns with this concept.

As hypothesized, PL resulted in a significantly higher accumulation of [^68^Ga]Ga-NODAGA-RGD within the infarct region at 5 days post MI. A longitudinal study in rats from Schwaiger´s group revealed highest focal tracer accumulation within the infarct around day 7 [20]. Subsequent studies confirmed correlation of tracer accumulation with newly formed vessels, which suggested the successful imaging of angiogenesis in the process of myocardial healing [18, 21]. Murry´s group identified a peak in granulation tissue formation and vascularization of the wound seven days after surgical permanent LAD occlusion in mice [32]. We have recently confirmed high expression of α_v_β_3_-integrin expression within the infarct in a mouse model on day 7 post-MI by [^68^Ga]Ga-NODAGA-RGD PET [7]. Vandelvelde et al. compared healing on a histological level after transient or permanent ligation of the LAD and found a higher angiogenic response in the I/R group [14]. Likewise, Murry ´s group hypothesized that interventions that accelerate the formation of granulation tissue will reduce ventricular remodeling [32]. Against this background, we have chosen day 5 and day 7 post-MI in the current study for imaging αvβ3-integrin expression, in order to capture the potential earlier peak of tracer accumulation in the I/R group. Yet, we found a minimal difference between the two time points for both groups at best.

Furthermore, we observed a positive correlation of EDV and tracer accumulation. This finding clearly shows, that the amount of α_v_β_3_-integrin expression corresponds with the extent of myocardial damage. One preclinical study has correlated RGD tracer accumulation within the infarct and ESV as an indirect measure of infarct size: Sherif et al. found that low ^18^F-RGD uptake after 7 days was an independent predictor of subsequent LV dilatation in rats [21].

Recent clinical studies have demonstrated the feasibility of PET imaging of α_v_β_3_-integrin expression using radiolabeled tracers containing the RGD motif after recent AMI: ^18^F-Fluciclatide [33], ^18^F-Galacto-RGD [22] and ^68^Ga-NODAGA-RGD [34, 35].

Importantly, in contrast to preclinical studies in rodents, AMI patients included in the above-mentioned studies received emergency coronary angiography with successful revascularization. Hence, LVEF was only mildly reduced at baseline reaching normal values at follow-up [33, 34]. In contrast, mice from the PL group in our study exhibited a massive decline of cardiac function with an average LVEF of approximately 30%.

Each of these studies report clear focal tracer accumulation within the injured myocardium. Three of these studies correlated tracer uptake with the functional or morphological defect. Whereas Jenkins et al. [33] found no correlation with infarct size, Makowski et al. [22] describe positive correlation of tracer uptake with infarct size and Nammas et al. report positive correlation of tracer uptake with LV-dysfunction [34].

Interestingly, two of these studies assessed cardiac functional parameters both at the time point of the PET study and after a follow-up period of 1 to 9 months.

Jenkins at al. found increased ^18^F-fluciclatide uptake in segments displaying functional recovery after 9 months.

Nammas et al. found that [^68^Ga]Ga-NODAGA-RGD predicted improvement in global longitudinal strain after 6 months.

Despite these promising findings, Nammas et al. conclude: “α_v_β_3_-integrin expression may provide information about the activation of the repair process after ischemic myocardial injury, but its utility as an imaging biomarker after human AMI remains uncertain.“

In summary, the existing clinical evidence confirmed findings from preclinical studies: imaging α_v_β_3_-integrin by the use of RGD-based PET tracers is feasible. The main challenge remains: is the α_v_β_3_-integrin a marker of a specific biological process, e.g. angiogenesis, in the healing myocardium?

In our study, [^68^Ga]Ga-NODAGA-RGD PET showed increased expression of αvβ3 integrin within the infarct area at day 5 and 7, a phase which corresponds to the formation of “granulation tissue” on a histological level. Going more into detail of the highly dynamic healing process, this phase corresponds to the transition of the proliferation to the maturation phase of the scar and involves inflammation, angiogenesis, extracellular matrix remodeling and scar formation [36, 37]. The interaction of macrophages, fibroblasts and endothelial cells with molecules from the extracellular matrix (ECM) is central in this process and takes place via different signaling pathways. Anti-inflammatory macrophages are key players orchestrating this process: they secrete factors that promote proliferation of both fibroblasts (TGFβ1) and endothelial cells (VEGF). Fibroblasts in turn start suppressing angiogenesis after 7 days, shifting the healing process towards the maturation phase [36]. Furthermore, fibroblasts serve as detectors of mechanical forces, in order to produce the appropriate collagen via specific receptors by which there are linked to ECM proteins, such as the α_v_β_3_ integrin [38]. Since cell-ECM interaction is essential for each involved cell type, the α_v_β_3_ integrin is expressed on macrophages, endothelial cells and fibroblasts [5, 6, 39].

In our study, the positive correlation of EDV and [^68^Ga]Ga-NODAGA-RGD uptake suggested a causal relationship between the extend of myocardial damage and the extent of α_v_β_3_ expression.

A recent study by *Yakota et al.* described a reciprocal feedback mechanism between extracellular matrix and the mechanosensitive receptor α_v_β_3_ integrin which drives fibroblast activation [38]. Hence, we wondered if the bulk of α_v_β_3_ expression in the context myocardial healing 5 days post MI, also reflects fibroblast activation.

In order to identify the cell population with the highest α_v_β_3_-integrin expression, we performed single nucleus transcriptomics at day 5 post MI in a separate set of experiments. Surprisingly, we found the highest rates of α_v_β_3_-mRNA expression in fibroblasts, yet not endothelial cells as hypothesized initially. This finding refutes the concept, that the bulk of α_v_β_3_ integrin is expressed on the tips of vessels sprouting into the provisional matrix of the healing scar. As a consequence, our data suggest that α_v_β_3_ integrin is not a valid target for imaging post-MI angiogenesis.

We also examined, if I/R results in a higher angiogenic response than PL as described by others based on histological data [14]. Flt1, Kdr, Flt4, and Tie1 are recognized markers of markers of angiogenesis after acute MI [15]. Except for Flt4, higher expression rates of the angiogenesis markers were indeed detected in the I/R group. Against our indirect observation, that the amount of α_v_β_3_ expression is predicted by the extent of myocardial damage, we suspected activated fibroblast to be the main source of α_v_β_3_ expression. Interestingly, we found markers of injury-activated fibroblasts – namely Fstl1, Col1a1, Col3a1 and Col5a1 - to be upregulated at day 5 post MI [16].

Sn-RNA sequencing revealed highest expression rates of these genes in the PL group, reflecting the formation of a significantly larger scar. Moreover, this coincidence of higher rates of injury-activated fibroblasts with higher rates of α_v_β_3_ expression in the PL group implies that NODAGA-RGD imaging reflects injury-activated fibroblasts, rather than angiogenesis in the context of myocardial healing post MI around day 5 post-MI. These findings are supported by immunohistochemistry showing a clear co-localization of α_v_β_3_ and Fstl1 within the infarct area.

From an imaging perspective there will certainly be an overlap with PET tracers targeting the fibroblast activation protein (FAP), which has been shown to be significantly upregulated during myocardial healing post-MI in both preclinical and clinical trials in contrast to quiescent fibroblasts in healthy individuals [40, 41].

In a rat model of permanent LAD ligation highest uptake of both [^68^Ga]Ga-FAPI-04 [42] and [^18^F]F-Galacto-RGD [20] was reported around day 6 and 7 post-MI throughout the whole infarct area. Hence, PET tracers targeting α_v_β_3_-integrin or FAP will most probably exhibit similar patterns of focal tracer accumulation from 5 to 7 days post-MI.

Likewise, clinical studies have shown similar patterns of tracer accumulation for both [^68^Ga]Ga-NODAGA-RGD [34] and [^68^Ga]Ga-FAPI-46 [41] around one week after acute myocardial infarction.

From a molecular perspective, both tracers bind highly specific to their target structure: namely α_v_β_3_-integrin and FAP respectively [3, 42]. Yet, from an in-vivo imaging perspective both targets are located within the same are, the granulation tissue of the healing scar.

Both tracers are certainly tools of utmost value, yet their clinical applicability for imaging post MI-healing will depend on a deeper understanding of the specific role in myocardial healing of both the α_v_β_3_-integrin and FAP.

In summary, we believe that our data will help substantially to understand the role of the α_v_β_3_-integrin, which is apparently expressed to the most significant extent in fibroblasts.

Hence, RGD-based tracers may not be the appropriate tool for monitoring angiogenesis even if the tracers per se have excellent imaging properties. In order use the clinical potential of [^68^Ga]Ga-NODAGA-RGD PET, future research should focus on deciphering patterns of beneficial healing in order to understand the actual role of α_v_β_3_-integrin in myocardial healing.

## Materials and methods

### Animal model

This study was conducted with the approval of the federal animal care committee of the Landesamt für Landwirtschaft, Lebensmittelsicherheit und Fischerei Mecklenburg-Vorpommern (LALLF) in Germany (reference number LALLF M-V 7221.3-1-032/20). All animal experiments were performed according to national guidelines in compliance with the German Animal Welfare Act and conform with Guide for the Care and Use of Laboratory Animals published by the US National Institutes of Health (NIH Publication No. 85 − 23, revised 1996), and every effort was made to minimize suffering. For our research, female 129S6/SvEvTac mice (age 160–180 days; weight 20.59 ± 1.50 g, range 18.00–24.75 g) were used in this study. These mice were bred and maintained in the animal facility of the University Medical Center Rostock and kept under specified pathogen-free conditions.

Myocardial infarctions were induced under anaesthesia with intraperitoneal injection of pentobarbital (50 mg/kg) und subcutaneous injection of fentanyl (0,2 mg/kg) as described before [7]. The left anterior descending coronary artery (LAD) was occluded either permanently (PL; *n*=−9) or transiently for 30 min (I/R; *n* = 9). In a sham group, thoracotomy and stitching the needle underneath the LAD without ligation was performed (SHAM; *n* = 8). [^68^Ga]Ga-NODAGA-RGD PET was performed at day 5 and 7 followed by cardiac MRI on day 28 post MI in every single animal. Mice were euthanized by cervical dislocation for histological analyses after the last scan under ongoing anaesthesia with isoflurane. Single nucleus RNA transcriptomics were performed in separate group 5 day after MI induction (PL: *n* = 4; I/R: *n* = 4, SHAM: *n* = 4). A separate group of 2 mice was used for immunofluorescence staining of fibroblast within remote and scar area (PL: *n* = 4).

### Tracer preparation

[^68^Ga]Ga-NODAGA-RGD was prepared in a manual synthesis: The activity from the GalliaPharm generator (Eckert & Ziegler) was trapped onto a PS-H + cartridge (Chromafix), which was eluted into the reaction vial (preloaded with 1 mL of 1.5 M HEPES) with a solution of 5 M NaCl/0.15% HCl (3 mL). Subsequently, 30 µL of 10 M NaOH was added, and the solution was mixed thoroughly. The precursor, 10 µg NODAGA-RGD trifluoroacetate (9805, ABX, Radeberg), was then added, and the reaction mixture was heated for 15 min at 90 °C. Afterwards, the solution passed through a preconditioned C18 cartridge (Strata-X RP-18, conditioned with 2 mL ethanol and 10 mL water), whereby the product was trapped. After rinsing with 2 mL water, the elution of the product was achieved with 3 mL of a 50% ethanol/water mixture.

In order to achieve an activity concentration of about 1 GBq/mL, all solvents of the synthesis product were removed via rotary evaporator at 60 °C. The dried product was dissolved in 100 µL of a 0.9% sodium chloride solution (Fa. B. Braun, Melsungen). The molar activity after this preparation was in the range of 8.8–13.7 MBq/nmol. The pH value of the final solution was 6. The activities were determined using a dose calibrator (MED Isomed 2010).

Analytical HPLC was performed using a CS MultoHigh 100 RP18 column (5 μm, 4 × 250 mm) on a Shimadzu HPLC pump and UV detector (220 nm) connected to a radiodetector. The mobile phase consisted of: A = water with 0.1% TFA, B = acetonitrile with 0.1% TFA, gradient: 0–0.5 min 10% B, 0.5–7.0 min 55% B, 7.0–30.0 min 55% B. Flow rate: 1 mL/min. Radiochemical identity and purity were verified for most batches via high-performance liquid chromatography by comparing the retention time of [^68^Ga]Ga-NODAGA-RGD (tR = 8.4 min) with that of [^69^Ga]Ga-NODAGA-RGD (9807, ABX, Radeberg, tR = 8.4 min). The observed purity measured by HPLC was in the range of 97.9–99.2%.

Additionally, thin-layer chromatography was performed on a Raytest miniGita Star using iTLC SG paper (Agilent) and a mixture of NH4OAc in water and methanol (1:1) as the mobile phase. The highest observed value for free 68Ga determined by iTLC was 0.24%.

### Positron-emission-tomography

Static PET/CT scans were performed on a small animal PET/CT scanner (Inveon MM-PET/CT, manufactured by Siemens Medical Solutions in Knoxville, TN, USA) according to established protocols. Mice were anaesthetized using isoflurane (4% for induction and 1.5–2.5% in O2 for maintenance during preparation and scanning). Approximately 15–20 MBq of [^68Ga]-NODAGA-RGD were injected intravenously via a custom-made micro catheter placed in a tail vein. Following a 30-minute uptake period, static images were acquired in the prone position for 45 min. During the PET scans, respiration was monitored, and the core body temperature of the mice was maintained at 38 °C using a heating pad. Whole-body CT scans were acquired for attenuation correction and anatomical land marking. PET data sets were corrected for random coincidences, dead time, scatter, and attenuation. Image reconstruction was performed using the three-dimensional iterative ordered-subset expectation maximization (3D-OSEM/OP-MAP) algorithm with the following parameters: four iterations (OSEM), 32 iterations (MAP), a target resolution of 1.7 mm, and a matrix size of 128 × 128. All data were decay-corrected to the time of injection. Image analysis of [^68^Ga]Ga-NODAGA-RGD uptake an Inveon Research Workplace (Siemens, Knoxville, TN, USA) was utilized, as described in a recent work by our group [7].

### Cardiac magnetic resonance imaging and analysis

The Cardiac magnetic resonance measurements were conducted using a 7-Tesla small animal MRI system (BioSpec 70/30) manufactured by Bruker BioSpin GmbH in Ettlingen, Germany under general anesthesia with isoflurane (4% for induction and 1.5–2.5% in O2 for maintenance during preparation and scanning). The system was equipped with a ^1^H transmit volume coil and a two-by-two receive-only surface coil array, both also manufactured by Bruker BioSpin. Images of the left ventricle for functional and morphological measurements were acquired using an IntraGate gradient-echo cine sequence (IntraGate Cine-FLASH) in five to seven short-axis planes covering the left ventricle. The following acquisition parameters were used: echo time (TE): 2.38 ms, repetition time (TR): 5.89 ms, flip angle: 15°, 14 frames per cardiac cycle, oversampling: 140, averages: 1, field of view (FOV): 29.4 × 25.2 mm, matrix size: 211 × 180, resolution in-plane: 0.14 × 0.14 mm, slice thickness: 1 mm, scan time per slice: 2 min. Body temperature of the animals was maintained by a water filled heating mat during the whole scan. Temperature of the water-filled heating mat was monitored and controlled by water bath with immersion circulator (HAAKE SC 100 and HAAKE S5P, Thermo Fisher Scientific, Schwerte Germany). Body temperature was continuously monitored by a small rectal temperature probe and maintained between 36 and 37 °C throughout the examination as previously ­ described [17]. Respiration cycles and Cardiac BPM were extracted from the Intragate navigator analysis. Cardiac function and morphology were assessed from the cine sequences using the freely available software Segment v4.0 R11044b (http://segment.heiberg.se) [43], as described in our recent work [17].

### Histological analysis und immunofluorescence staining

Following the last scan, 100 µg/100 µl biotinylated Lycopersicon esculentum (tomato lectin) (Vector Labs; Burlingame, CA) was injected through a microcatheter placed in the tail vein of the mice for labeling vascular elements [44]. After 15 min hearts were excised and flash frozen in liquid nitrogen with Tissue-Tek^®^ O.C.T. Compound (Sakura Finetek, Alphen aan den Rijn, Netherlands) with liquid nitrogen. Frozen hearts were cryosectioned into 6 μm axial sections at four different levels from the apex to the base.

For fibrosis estimation, two contiguous sections representing the middle section of the heart were stained with Fast Green FCF (Sigma-Aldrich) and Sirius Red (Chroma Waldeck GmbH & Co. KG, Münster, Germany) and assessed using computerized planimetry (Axio Vision LE Rel. 4.5 software, Carl Zeiss AG, Oberkochen, Germany). The left ventricle was then split into six segments and relative fibrotic area was quantified with Fiji software (Ver. 2.90) [45] and the Trainable Weka Segmentation plugin [46].

For capillary density assessment immunostaining with a polyclonal goat anti-biotin primary antibody (Vector Laboratories), followed by incubation with a donkey anti-goat Alexa Fluor^®^ 488 conjugated secondary antibody (Thermo Fisher Scientific) was used.

Cardiomyocytes membranes were stained with Wheat Germ Agglutinin (WGA) conjugated with Alexa Fluor^®^ 488 (Thermo Fisher Scientific) for assessment of cell size. Nuclei were visualized and counterstained using DAPI (Sigma-Aldrich).

Fluorescence images were acquired using a Zeiss ELYRA PS.1 LSM 780 confocal imaging system with a 20x/0.8 objective.

The number of capillaries in the infarcted scar tissue and uninjured left ventricular myocardium was quantified using Fiji. Three randomly chosen fields (each 0.05 mm²) were analyzed in both the scarred and remote regions, and the number of capillaries present in each field was counted.

To assess myocardial hypertrophy, the area of 10 cardiomyocytes was measured in each of three randomly selected fields (0.05 mm²) in both scar and infarcted regions, therefore a total of 60 cells per mouse from a total of 6 fields were evaluated. To ensure the most accurate measurement of cardiomyocyte area, only cells with a visible nucleus at the center were included in the analysis.

For assessing regional expression of α_v_β_3_ in the context of activated fibroblasts and vessels (Fstl1 and CD31), heart slices from mice 5 days post permanent ligation of the LAD were fixed with 2% paraformaldehyde for 15 min, followed by permeabilization using 0.2% Triton X-100 (both from Sigma-Aldrich) for 5 min. To avoid nonspecific binding, cells were incubated with 1% bovine serum albumin (BSA). Primary antibodies targeting FSTL1 (R&D Systems, AF1738, dilution 1:100), Integrin (Abcam, ab179473, dilution 1:100) CD31 (R&D Systems, AF3628, dilution 1:100) were applied overnight at 4°. Following several washing steps, tissue slices were incubated with corresponding secondary antibodies donkey anti-rabbit Alexa Fluor568 and donkey anti-goat Alexa Fluor 647 (Thermo Fisher, dilution 1:300) for 2 h at room temperature. Nuclei were visualized by DAPI staining. Microscopy was performed using a Zeiss ELYRA LSM 780 imaging system with 40 × alpha 1.46 Plan apochromat objective with oil immersion (Zeiss, Oberkochen, Germany). For image acquisition, 4 × 4 or 5 × 5 tiles scans were recorded as z-stack sequences.

We quantified the co-expression of α_v_β_3_ and FSTL1 using an ROI-based approach to assess their presence within the same cell. ROIs were defined based on nuclear segmentation and expanded to approximate the cell area. After background subtraction, only ROIs with positive intensity values for both α_v_β_3_ and FSTL1 were considered to indicate co-expression of the two proteins within the same cell.

### Isolation of nuclei and sequencing

Whole hearts were harvested from 4 mice per group (PL: *n* = 4; I/R: *n* = 4, SHAM: *n* = 4) five days after MI induction after cervical dislocation. Hearts of the respective groups were pooled and nuclei were isolated according to the manufacturer’s protocol followed by single-nucleus sequencing as described in detail in [11].

### Computational data analysis of snRNA-Seq data

In general, the scRNA-Seq data analysis involved quality control, normalization, confounding factor identification, dimensionality reduction, and cell – gene level analysis, as previously described [10]. In particular, the data analysis steps were as follows: Processing of the fastq data was conducted by using the Cell Ranger Software (v.6.1.2) to the mm10 genome (Ensembl release 103) index, annotated via GTF file and grouped by barcodes and UMIs resulting in a feature-barcode matrix. Downstream analysis was performed using Seurat (v.4.2.0) [47]. After following the standard pipeline of normalization (SCT) [48], finding variable features, scaling, and dimensionality reduction by principal-component (PC) and visualization via Uniform Manifold Approximation and Projection (UMAP), the three datasets were merged in a single Seurat object to correct for batch effects and allow for an integrative analysis with the upstream processing algorithm Harmony (v.0.1) [49]. The integrated dataset was then used for UMAP clustering utilizing the formerly generated Harmony embedding’s.

Since annotation of clusters is still considered as a domain specific effort, we utilized multiple approaches to account for the individual complexity of the dataset. First, sets of known marker genes, as well as recently identified cell cluster markers by other groups and our own are applied as indicated in the provided computational script [12, 13, 50]. In addition, the top 100 transcripts per cluster (if available) and identified cell cluster markers (via in-built Seurat functions) from our dataset served as basis for the annotation of all identified clusters. The median of the respective cell clusters has been computed via the in-build stat_summary function of the ggplot2 package (v.3.4.2).

### Statistical analysis

In this study, descriptive statistics for continuous variables are reported as mean ± standard deviation. Statistical analysis was conducted using SigmaPlot (Systat Software, San Jose, CA). A p-value less than 0.05 was considered statistically significant. To assess the normality of the data, we used the Shapiro-Wilk test. In case of normally distributed parameters, we employed one-way ANOVA for group differences among the PL, I/R, and SHAM groups, and post-hoc testing with correction for multiple comparisons using the Tukey-Kramer method. If the normality assumption was violated, we used ANOVA on ranks, followed by the Dunn’s method.

For correlation between 2 continuous variables, Spearman´s rank coefficient was used (r_s_).

We presented data distribution using box and whisker plots that show the median, 25th and 75th percentile, maximum and minimum values, or, violin plots. R and the ggplot2 package were utilized for producing figures [51, 52].

## Supplementary Information

Below is the link to the electronic supplementary material.

Supplementary file1 (DOCX 14.0 MB)

## Data Availability

The datasets generated during and/or analysed during the current study are available from the corresponding author on reasonable request.
