# Supplementary material for: [68Ga]Ga-NODAGA-RGD post MI reflects activated fibroblasts rather than angiogenesis
Source: Eur J Nucl Med Mol Imaging. 2025 Jul 29;53(2):1064–80. doi: 10.1007/s00259-025-07489-4 (PMC12830458; doi:10.1007/s00259-025-07489-4)
Supplement: Supplementary file 1 — Supplementary file1 (DOCX 14.0 MB) [file 259_2025_7489_MOESM1_ESM.docx]

***Regional Properties of the Left Ventricle***

Global LV dimensions and derived functional parameters, such as EDV and LVEF, are widely accepted as surrogate markers of LV remodeling and cardiac function in both clinical and preclinical settings. Nevertheless, these functional parameters do not provide information about properties of the scar itself. Hence, we evaluated regional scar properties based on a cine CMR approach, recently developed by us [17].

The septal myocardium was thicker in both PL and IR compared to the SHAM group, reaching a level of statistical significance in the PL group only. (SHAM: 0.76 ± 0.07 mm, PL: 0.90 ± 0.15 mm, IR: 0.82 ± 0.06 mm; SHAM vs PL: p=0.027).

Relevant thinning of the scar – represented by the lateral wall thickness - was present in the PL group only (SHAM: 0.83 ± 0.11 mm, PL: 0.36 ± 0.11 mm, IR: 0.77 ± 0.10 mm; SHAM vs PL: p<0.001) (*Supplemental Figure 1, 2*).

Likewise, wall thickening as a measure of radial contractility, was significantly reduced in the PL group in the lateral segments of the LV wall, corresponding to the scar area (SHAM: 0.35 ± 0.09 mm, PL: -0.02 ± 0.04 mm, IR: 0.30 ± 0.07 mm; SHAM vs PL: p<0.001). Wall thickening in the septal wall seemed impaired in the PL group, yet not to a statistically significant extent (SHAM: 0.36 ± 0.06 mm, PL: 0.24 ± 0.12 mm, IR: 0.32 ± 0.10 mm). (*Supplemental Figure 1, 2*).

***Supplemental Figure 1:* Regional Properties of the LV visualized as bulls-eye plots.** Representative images of SHAM, PL and IR animals. Upper row: end-diastolic wall thickness. Lower row: wall thickening. The inner ring represents the apical slice, the outer ring the basal slice. Each slice was divided into six segments as visualized. The septal wall is represented by the respective segments 2 and 3, the lateral wall by the respective segments 5 and 6.


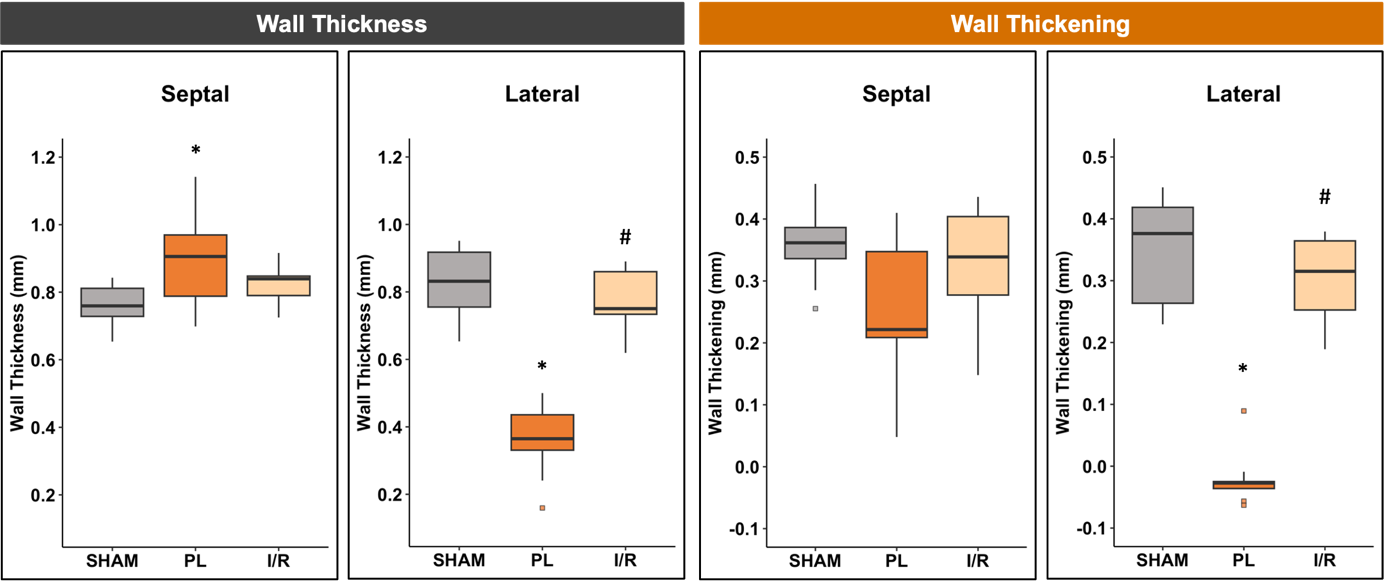


***Supplemental Figure 2:* Regional wall analysis of short-axis slices 3–5.** Wall thickness was assessed in end-diastolic images. Wall thickening is the difference between end-systolic and end-diastolic wall thickness. Boxplots show medians (center lines), 25th and 75th percentiles (box limits), and whiskers (1.5 IQR); outliers are squares. One-way ANOVA followed by Tukey’s post hoc was used to compare experimental groups. * p < 0.05 compared to control group. # p < 0.05 compared to PL group. SHAM: n = 8; PL: n = 9; I/R: n = 9.

Capillary density of the left ventricle was quantified by tomato lectin staining within septal and lateral segments separately. Capillary density was dramatically reduced in the lateral segments (scar) in the PL group compared to the SHAM group (PL vs SHAM: p<0.001). Similar observations were made in the septal segments (PL vs SHAM: p=0.027) (*Supplemental Figure 3*).

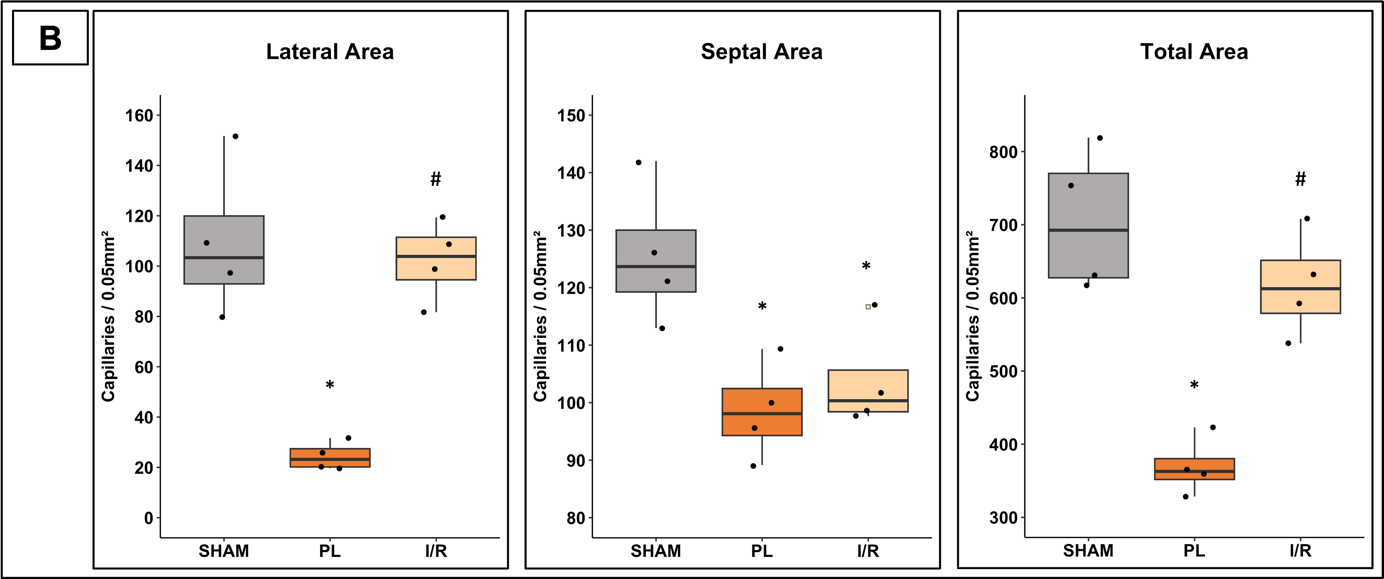


***Supplemental Figure 3: Capillary Density 4 Weeks post-MI. A*** Capillary density was assessed by fluorescence microscopy using tomato lectin and DAPI for cell nuclei. ***B*** Comparison of capillary density in the septal, lateral and total LV area. Boxplots show medians (center lines), 25th and 75th percentiles (box limits), and whiskers (1.5 IQR). Outliers are squares; individual data points are dots. One-way ANOVA followed by Tukey’s post hoc was used to compare experimental groups. * p < 0.05 compared to SHAM group. # p < 0.05 compared to PL group. SHAM: n = 4; PL: n = 4; I/R: n = 4. Scale bar: 100 µm.

Cross-sectional cardiomyocyte size, as a measure of hypertrophy, was quantified by WGA staining of the cell membrane. As expected from *in-vivo* CMR data, only PL resulted in a statistically significant hypertrophy of cardiomyocytes in the whole LV as well as in the lateral and septal segments when assessed separately (lateral: SHAM: 250.89 ± 17.21 µm², PL: 386.08 ± 38.61 µm², IR: 268.18 ± 14.01 µm²; p = 0.002; septal: SHAM: 252.50 ± 11.71 µm², PL: 324.58 ± 22.98 µm², IR: 254.40 ± 18.19 µm²; p = 0.002) (*Supplemental Figure 4*).

***
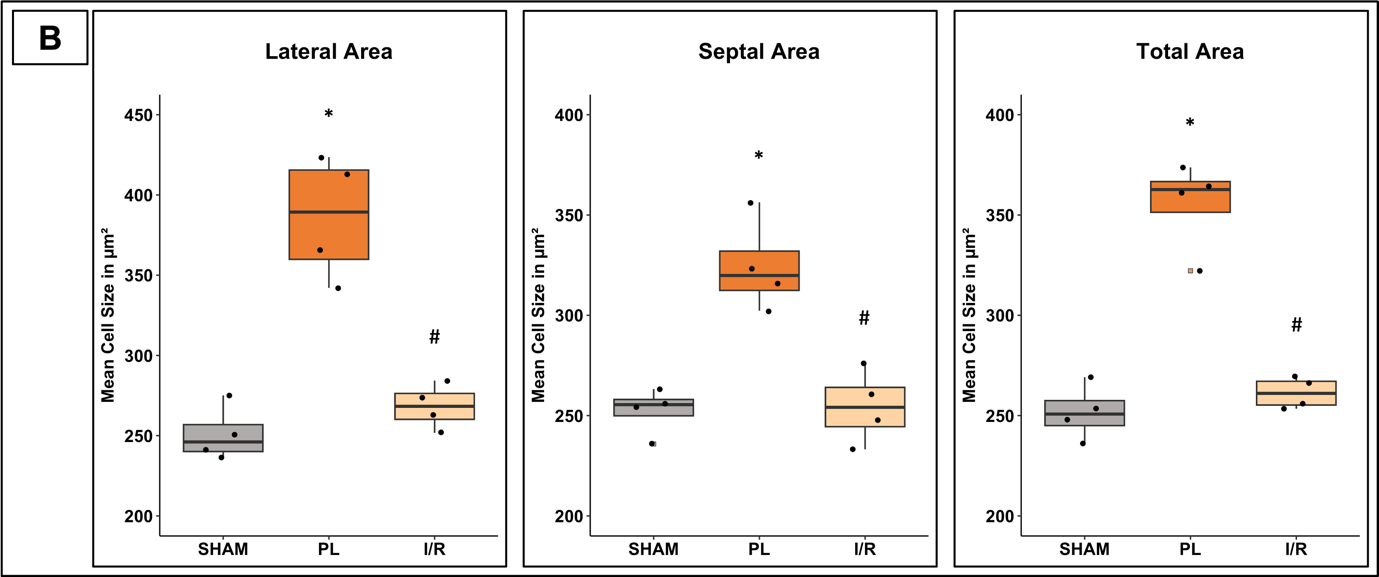
***

***Supplemental Figure 4:* Average cardiomyocyte size 4 weeks post-MI. A** Cell membrane labeled by FITC-conjugated WGA (green) and cell nuclei by DAPI (blue). **B** comparison of average cardiomyocytes size in the septal, lateral and total LV area. Boxplots show medians (center lines), 25th and 75th percentiles (box limits), and whiskers (1.5 IQR). Outliers are squares; individual data points are dots. One-way ANOVA followed by Tukey’s post hoc was used to compare experimental groups. * p < 0.05 compared to SHAM group. # p < 0.05 compared to PL group. SHAM: n = 4; PL: n = 4; I/R: n = 4. Scale bar: 100 µm.
